# En bloc right hemicolectomy with pancreatoduodenectomy for right-sided colon cancer invading duodenum

**DOI:** 10.1186/s12893-021-01286-0

**Published:** 2021-06-29

**Authors:** Xiao-Luan Yan, Kun Wang, Quan Bao, Hong-Wei Wang, Ke-min Jin, Jun-Yun Wang, Bao-Cai Xing

**Affiliations:** 1 Key Laboratory of Carcinogenesis and Translational Research (Ministry of Education/Beijing), Hepatopancreatobiliary Surgery Department I, Peking University Cancer Hospital & Institute, Beijing, China; 2 CAS Key Laboratory of Genome Sciences and Information, Beijing Institute of Genomics, Chinese Academy of Sciences, Beijing, China; 3 GloriousMed Holdings Co., Ltd., No.11, Lane 100, Banxia Road, Pudong New Area, Shanghai, China

**Keywords:** En bloc resection, Pancreatoduodenectomy, Locally advanced right-sided colon cancer right hemicolectomy, Survival

## Abstract

**Background:**

En bloc right hemicolectomy with pancreatoduodenectomy (RHCPD) is the optimum treatment to achieve the adequate margin of resection (R0) for locally advanced right-sided colon cancer with duodenal invasion. Information regarding the indications and outcomes of this procedure is limited.

**Method:**

In this retrospective study, 2269 patients with right colon cancer underwent radical right colectomy between October 2010 and May 2019, in which 19 patients underwent RHCPD for LARCC were identified. The overall survival (OS), disease-free survival (DFS), operative mortality, postsurgical complications, gene mutational analysis, and prognostic factors were evaluated. Survival was estimated using Kaplan–Meir method.

**Results:**

Of these 19 patients who underwent LARCC, the OS was 88%, 66%, and 58% at 1, 3, and 5 years. The DFS was 72%, 56%, and 56% at 1, 3, and 5 years. The median operative time was 320 min (range: 222–410 min), and the median operative blood loss was 268 mL (range: 100–600 mL). The OS was significantly better among patients with well-differentiated tumor, N0 stage, and high microsatellite instability (MSI) and in patients who received adjuvant chemotherapy. The major postoperative complications occurred in 8 patients (42%), with pancreatic fistula (PF) being the most common. On the basis of the univariate analysis, poorly differentiated tumor, regional lymph node dissemination, MSI status, and no perioperative chemotherapy were the significant predictors of poor survival (*P* < 0.05).

**Conclusions:**

This study suggests that RHCPD is feasible and can achieve complete tumor clearance with favorable outcome, particularly in patients with lymph node-negative status.

**Supplementary Information:**

The online version contains supplementary material available at 10.1186/s12893-021-01286-0.

## Background

Colorectal cancer (CRC) ranks the third among malignancies in the world, and the mortality rate of patients with advanced CRC is high [1–3]. Adjacent organ invasion is found to be 5–24% in CRC patients [4, 5], and carcinoma of the right colon rarely invades adjacent viscera, the incidence of which is reported to be 0.9–2.6% [5–7]. Surgery is considered the first choice for CRC if possible [3]. Non-radical resection and blunt mobilization of colon cancer from adherent organs are associated with tumor recurrence, and the prognosis of such patients is poor [8–10]. En bloc resection is the curative resection for locally advanced CRC that has adhered to and/or invaded adjacent structures without distant metastasis. LARCC (locally advanced right-sided colon cancer) can involve the duodenum, pancreas, and other organs. Under such conditions, it is necessary to perform multivisceral or extended resection to achieve tumor negative margin of resection (R0).

*We believe that* radical RHCPD (right hemi-colectomy with pancreatoduodenectomy), first reported in 1953, is the preferred choice to achieve R0 resection of LARCC [11–13]. Despite the complexity of RHCPD, acceptable mortality and morbidity rates have been reported in several studies [7, 11, 14]. Nevertheless, clinicopathological findings and long-term outcomes of LARCC patients treated by RHCPD are rarely reported [5, 12, 15–18]. In China, the histologic findings and long-term survival of LARCC treated by RHCPD are also rarely reported [5, 12, 19]. Moreover, the potential relationship between the clinicohistologic-genetic status and prognosis is unknown. In this retrospective study, we aimed to evaluate RHCPD in achieving radical tumor removal of LARCC patients with favorable outcomes and to identify prognostic factors of such patients with malignant involvement of adjacent organs as well as their gene expression and pathologic characteristics.

## Methods

### Patient characteristics

Patients who were diagnosed with primary right-sided colon cancer and accepted radical right colectomy between October 2010 and May 2019, at the Beijing Cancer Hospital, were retrospectively analyzed. The data of demographics, estimated blood loss, duration of surgery, adjuvant and/or neoadjuvant chemotherapy, tumor pathology, mortality, morbidity, and long-term outcomes were collected. Inclusion criteria: (1) no metastasis revealed by preoperative imaging; (2) potential curative resections; (3) colon carcinoma confirmed histologically; (4) T4 malignancy either to pancreas or duodenum revealed by biopsy; (5) available radiologic data at follow-up in our institution. Exclusion criteria: (1) local recurrent tumor; (2) distant metastasis; (3) secondary involvement of the pancreatic head and/or duodenum other than direct infiltration. The protocol was approved by the ethics committee of Beijing Cancer Hospital, and all the patients signed the written informed consent. The 1964 Helsinki declaration and its subsequent amendments were followed.

### Diagnosis and indications for RHCPD

Local tumor infiltration was evaluated using preoperative computed tomography (CT). Loss of the fat space between the duodenum and the recurrent tumor, protrusion of a nodular mass into the duodenum, or involvement of the head of the pancreas indicated that the tumor involved the duodenum and/or the head of the pancreas. CEA (carcinoembryonic antigen) and CA19-9 (cancer antigen 19-9) levels of all patients were detected before surgery. Colonoscopy and histopathologic determination of the tumor were performed before surgery to confirm colon cancer justifying the need for RHCPD. Below are indications for RHCPD: (1) histologic confirmation of colon carcinoma before surgery; (2) impossible to dissociate the tumor from the pancreas and/or duodenum with gentle mobilization; (3) feasible radical resection according to preoperative evaluation and no distant metastasis; and (4) no severe comorbidity able to tolerate a radical multivisceral excision [5, 16, 17].

### Surgical procedure for RHCPD

#### Resection

We first performed a Cattell–Brasch maneuver, and an extended Kocher maneuver was then used to mobilize the duodenum fully [14]. We assessed the amount of infiltration into the pancreas and/or and duodenum and resectability of LARCC after complete mobilization of the right colon and duodenum without removal of the adherent organs. After R0 resection based on the standard procedures, RHCPD was performed. If the SMV (superior mesenteric vein) and/or portal vein was involved, the mesentericoportal vein was also dissected followed by an end-to-end anastomosis.

#### Reconstruction

Following the methods of modified Child’s reconstruction, we performed reconstruction with an end-to-side pancreaticojejunostomy or pancreaticogastrostomy, which depended on the pancreatic duct. During pancreatojejunostomy procedures, the stent of the pancreatic duct was used, which is an intraluminal stent left to fall out on its own. Stapled side-to-side anastomosis of the ileum and transverse colon was introduced for the bowel reconstruction. After the procedures above, we inserted rubber drains near the pancreatic and biliary anastomoses, and the incision at the abdominal wall was sutured [5, 14, 16].

### Pathology and gene testing

Based on the classification of AJCC (American Joint Committee of Cancer), the tumor stage was evaluated [20]. Postoperative complications, such as DGE (delayed gastric emptying), PF (pancreatic fistula), and intraabdominal abscess, were evaluated. Postoperative PF and DGE were defined based on the International Study Group on Pancreatic Fistula Definition and the International Study Group of Pancreatic Surgery [21–23], respectively. If infected pus and fluid inside the abdominal cavity was collected, intraabdominal abscess was diagnosed (Additional file 1).

Tumor cellularity was determined using formalin-fixed, paraffin-embedded blocks. Tumors were macrodissected for removal of normal tissues, and samples containing > 20% neoplastic cells were harvested. Sample preparation, exome capture, library construction, bioinformatics analyses and NGS (next-generation sequencing) of cancer and normal samples were carried out at GloriousMed Holdings Co., Ltd. (Pudong New Area, Shanghai). NRAS, KRAS, BRAF, HER4 and HER2 mutations were determined using NGS.

Microsatellite instability (MSI) Analysis System Version 1.2 (Promega, Madison, WI) was used to determine MSI (microsatellite instability), which included five pseudomonomorphic mononucleotide repeats (NR-24, NR-21, BAT-26, MONO-27, and BAT-25). Over 2 of 5 altered markers indicated high MSI (MSI-H).

### Follow-up

All patients were followed up after surgery, with a 3-month interval during the first 2 years, then 6-month interval during the subsequent 3 years, and at least 1-year interval thereafter. During each follow-up, CEA and CA19-9 testing, abdominal CT or ultrasound, chest radiography, and physical examination were carried out. The standard protocol was followed during the follow-up [24]. Patients underwent colonoscopy annually at outpatient clinics.

### Primary and secondary outcomes

Overall survival (OS) was deemed as the primary endpoint. The secondary endpoints included 30-day postoperative mortality, postsurgical complications, DFS (disease-free survival), prognostic factors and gene testing results.

### Statistical analysis

SPSS v.16.0 software (provided by SPSS, Chicago, IL) was used for data analysis. The median (range) was used express numerical data. The KM (Kaplan–Meier) method was used to assess the survival rate. Univariate analysis was performed to assess prognostic variables, and *P* < 0.05 indicated statistical significance.

## Results

### Patients characteristics

Between October 2010 and May 2019, 2269 patients with primary right-sided colon cancer underwent radical RC at the Beijing Cancer Hospital. Among them, 19 patients (12 men and 7 women) underwent RHCPD for LARCC with direct infiltration into the duodenum and/or pancreas. The median age of the patients was 60 years (range, 35–75 years). The tumors were located in the ascending colon (3 patients) and hepatic flexure (16 patients). Direct tissue invasion by the tumor into the duodenum was observed in all the patients, pancreas in 4 patients, the liver in 2 patients, and the SMV in 1 patient. The median preoperative CEA was 5.7 ng/mL (range: 0.9–18.5 ng/mL), and CA19-9 was 43.2 IU/mL (range: 0.5–228.4 IU/mL). During presentation, 13 patients had anemia, and 8 had abdominal pain. Other prominent symptoms were history of significant weight loss (n = 7), abdominal distension (n = 5), and vomiting (n = 5).

### Overall survival and disease-free survival

**Fig. 1:**
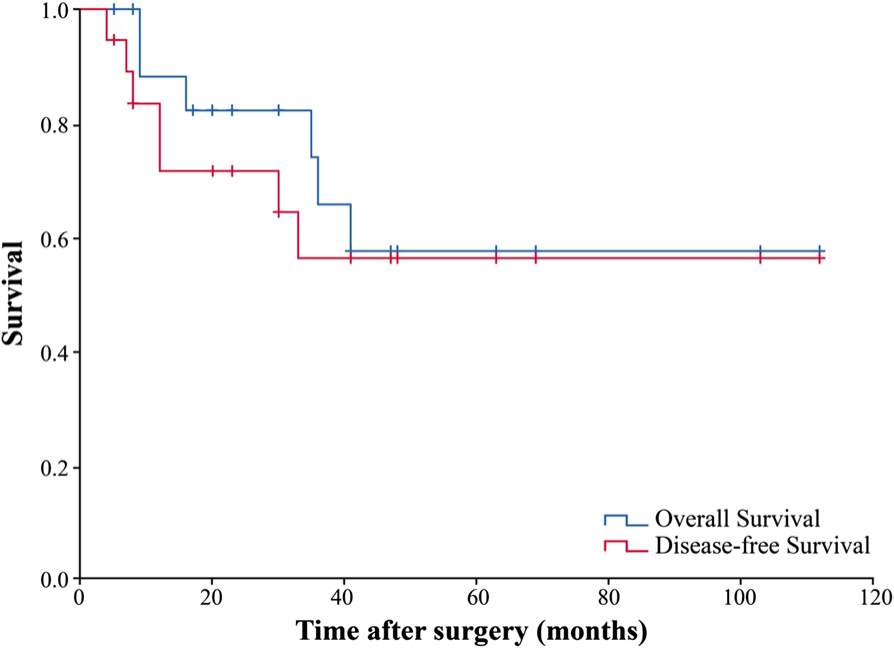
Overall survival and disease-free survival in overall patients

The median follow-up time was 39 moths (range: 5–112 months). During the follow-up period, 6 patients died from abdominal or liver metastases. The median survival time was 76 months (95% CI 53.3: 98.3). The 1-, 3-, and 5-year OS was 88%, 66%, and 58%, and DFS was 72%, 56%, and 56% respectively (Fig. 1). Among the 13 patients who were still alive at the last follow-up, 4 survived > 5 years with no recurrence (Cases 2, 3, 7, and 8).

**Fig. 2:**
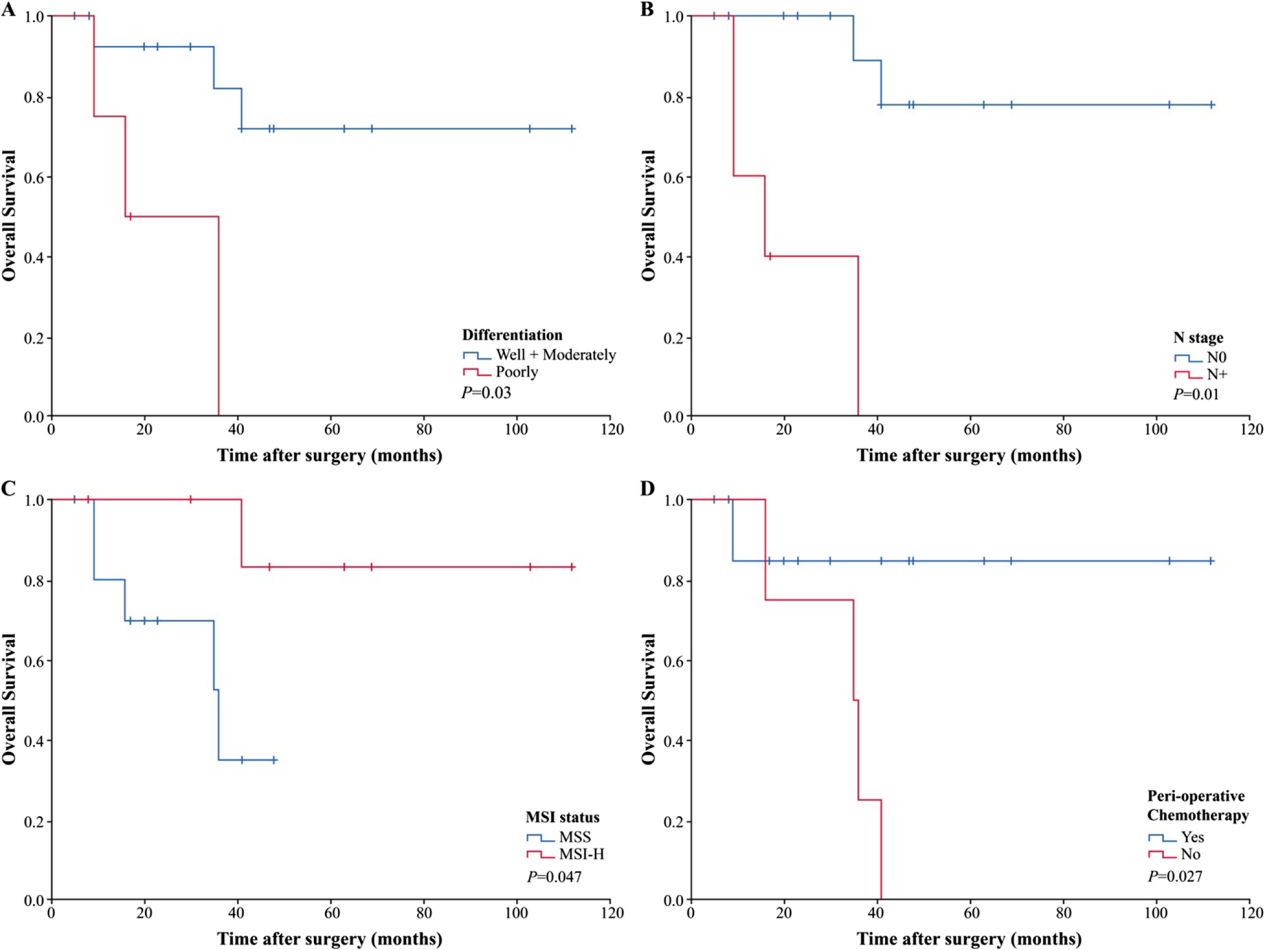
Overall survival KM curves in patients of various groups. **A** Patients with well- or moderately differentiated tumor versus poorly differentiated tumor. **B** Patients with N0 stage versus N+ stage. **C** Patients with MSS versus MSI-H. **D** Patients with adjuvant chemotherapy versus no chemotherapy. *MSI-H* microsatellite instability-high, *MSS* microsatellite stable

On the basis of the KM-survival curves between various groups, the survival was significantly better among patients with well- or moderately differentiated tumor (*P* = 0.03) (Fig. 2A), N0 stage (*P* = 0.01) (Fig. 2B), MSI-H (*P* = 0.047) (Fig. 2C) and in patients who received chemotherapy (*P* = 0.027) (Fig. 2D).

### Postoperative complications and treatment

The treatments and outcomes data of the patients are listed in Table 1. The median operative time was 320 min (range: 222–410 min), and the median operative blood loss was 268 mL (range: 100–600 mL). Blood was transfused intraoperatively in 11 patients with an average amount of 2.5 U (range: 2–6 U), also due to preoperative anemia. The postoperative hospital stay was 23.5 days (range: 11–45 days). None of the patients died during the postoperative 30 days.

**Table 1:** Clinical characteristics and survival outcome of the study patients with LARCC

Case	Sex	Age (years)	Site of colon cancer	Adjacent organ infiltration on preoperative CT	Preoperative therapy	Operation	LOS (days)	Complications	Adjuvant therapy	Survival (months)	Status	Cause of death
1	M	48	ASC	Du + Pa	–	RHCPD	18	PF(C), IAA	–	35	Dead	Recurrence
2	F	44	HF	Du + Pa + SMV	FOLFOX4	RHCPD + SMVR	18	–	Capecitabine	112	Alive	
3	M	48	HF	Du + L	–	RHCPD	22	PF(B), IAA	mFOLFOX6	103	Alive	
4	M	60	HF	Du	–	RHCPD	45	IB	–	16	Dead	Recurrence
5	M	75	HF	Du	–	RHCPD	26	PF(B), IAA	–	36	Dead	Recurrence
6	M	62	ASC	Du	–	RHCPD	21	PF(A)	XELOX	9	Dead	Recurrence
7	F	36	HF	Du	mFOLFOX6	RHCPD	15	–	FOLFIRI	69	Alive	
8	F	65	HF	Du	–	RHCPD	16	PF(A)	XELOX	63	Alive	
9	M	45	HF	Du	–	RHCPD	21	PF(B), IAA	FOLFOX4	9	Dead	Recurrence
10	M	73	HF	Du	–	RHCPD	36	BF	–	41	Dead	Recurrence
11	M	35	HF	Du	–	RHCPD	15	PF(A)	XELOX	48	Alive	
12	M	65	HF	Du + Pa	–	RHCPD	26	PF(A)	XELOX	47	Alive	
13	F	46	HF	Du	–	RHCPD	20	BF, IAA	XELOX	41	Alive	
14	M	54	HF	Du + L	FOLFOXIRI + BEV	RHCPD	43	Ileus	FOLFOXIRI + BEV	30	Alive	
15	M	63	ASC	Du	–	RHCPD	26	–	XELOX	23	Alive	
16	F	42	HF	Du + Pa	XELOX + BEV + Pem	RHCPD	24	PF(A)	XELOX + BEV + Pem	20	Alive	
17	M	64	HF	Du	–	RHCPD	11	–	XELOX	17	Alive	
18	F	66	HF	Du	–	RHCPD	25	PF(A)	XELOX	8	Alive	
19	F	65	HF	Du	–	RHCPD	13	–	XELOX	5	Alive	

ASC, ascending colon; BEV, bevacizumab; BF, biliary fistula; Du, duodenum; F, female; FOLFIRI, folinic acid, fluorouracil, plus irinotecan hydrochloride; FOLFOX, folinic acid, fluorouracil, plus oxaliplatin; HF, hepatic flexure; IAA, intraabdominal abscess; IB, intraabdominal bleeding; LOS, length of stay; L, liver; m, modified; M, male; OBL, operative blood loss; Pa: pancreas; Pem, pembrolizumab; PF, pancreatic fistula; RHCPD, right hemicolectomy with pancreatoduodenectomy; SMV, superior mesenteric vein; XELOX, capecitabine plus oxaliplatin; SMVR, superior mesenteric vein resection

Major postoperative complications occurred in 8 patients (42%) with clinically significant PF being the most common. No patient had DGE, ileocolic, gastrojejunal, or jejunojejunal anastomotic leak. A total of 15 patients received adjuvant chemotherapy, including XELOX (9 patients), FOLFOX4 (1 patients), mFOLFOX6 (1 patient), FOLFIRI (1 patients), Capecitabine (1 patients), FOLFOXIRI + Bevacizumab (1 patients), and XELOX + Bevacizumab + Pembrolizumab (1 patients), in which 4 patients received neoadjuvant chemotherapy. (Table 1).

### Pathologic findings and genetic testing

Tumors were classified as well-differentiated adenocarcinoma (3 patients), moderately differentiated adenocarcinoma (12 patients), and poorly differentiated adenocarcinoma (4 patients) based on histologic findings. According to the AJCC classification system, 14 patients were N0 staged, 2 as N1b, 1 as N2a and 2 as N2b. All of the tumors had clear resection margins (R0). In mutation testing, 11 patients were K-Ras mutant, 1 patient was B-Raf V600E mutant, 2 were Her-2 mutant, and none of the patients were N-Ras mutant as identified by using NGS. A total of 8 patients were identified as MSI-high status.

### Prognostic factors for overall survival

On the basis of the univariate analysis, tumor differentiation, N stage, MSI status, and adjuvant chemotherapy were the significant prognostic factors (*P* < 0.05) (Table 2).

**Table 2:** Uni-analyses of factors associated with overall survival

Prognostic factors		Univariable P
Age, years	< 60/≥ 60	0.340
Sex	Male/Female	0.900
Preoperative CEA	< 5 ng/mL/≥ 5 ng/mL	0.877
Preoperative CA19-9	< 37 kU/L/≥ 37 kU/L	0.646
Operative time	< 320 min/≥ 320 min	0.509
Operative blood loss	< 400 mL/≥ 400 mL	0.933
Major complications	I/II–IV	0.748
Tumor differentiation	Poorly/well + moderately	0.030
N stage	N0/N+	0.010
MSI status	MSI-H/MSS	0.047
K-Ras	Wild/mutant	0.888
B-Raf	Wild/mutant	0.771
Her-2	Wild/mutant	0.635
Adjuvant chemotherapy	Yes/no	0.027

*CEA*, Carcinoembryonic antigen, *CA19-9* cancer antigen 19-9, *MSI-H* microsatellite instability-high, *MSS* microsatellite stable

## Discussion

Right-sided CRC invading duodenum and/or pancreas is a rare condition [6, 23, 25], and only a few studies have reported adjacent-organ resection [4, 6, 7, 14–16, 25–29]. In our study, among 2269 patients with primary right-sided CRC who underwent radical RC screened, only 19 patients (12 men and 7 women) underwent en bloc RHCPD for LARCC with direct infiltration into the duodenum and/or pancreas. The patients who underwent duodenal resection with correction by direct suture or pedicled ileal flap were excluded because of the poor outcome and high rate of morbidity and mortality. Cirocchi et al. summarized the results of 15 previous studies and reported a 5-years overall survival of 52% after en bloc pancreaticoduodenectomy plus right hemicolectomy vs. 0 and 25% in case of duodenal resection with correction by direct suture or pedicled ileal flap, respectively [8]. We agreed with the above views and tried to avoid local duodenectomy in clinical practice, LARCC once confirmed, all adhesions between tumor and adjacent organs should be considered as malignant invasion due to 33% to 84% malignant invasion on pathologic examination [4, 15] and should not be separated as there exist a risk of tumor recurrence rate of 90% to 100% [9, 10]. In our study, en bloc resection was performed, and adhesions were verified as malignant only after histopathologic examination.

Right-sided CRC invading duodenum and/or pancreas was considered to have poor outcomes and unresectable in the earlier days. However, recent studies have reported a promising prognosis, with a 5-year survival rate ranging from 21 to 55% in patients with LARCC invading adjacent organs undergoing en bloc multivisceral resection [4, 5, 12, 15, 19]. Similarly, in our study, all the patients who underwent curative RHCPD achieved good outcomes, with 1-, 3-, and 5-year OS rate of 88%, 66%, and 58%, whereas DFS rates of 72%, 56%, and 56% respectively. Comparatively higher OS rate in our study is probably because of the fact that even though all the patients were staged T4b, regional lymph node dissemination of the cancer may not be that advanced because only 5 out of 19 patients were lymph node positive (N2a and N1b). In addition, some colon cancers exhibits locally aggressive invasion instead of distant spread [4, 15, 27, 29]. According to the previous reports, 25% to 60% of right colon carcinoma that invaded the adjacent duodenum or pancreas do not have lymph node metastasis [4, 6, 15, 27, 29]. Furthermore, Saiura et al. reported significantly longer survival in patients with node-negative status than node-positive patients [15]. Similarly, in our study, the survival of patients with node-positive had short survival (< 3 years) at the time of last follow-up Meanwhile, the 3- and 5-year OS of 14 patients with N0 were 89% and 78%, respectively, among them 4 patients survived for > 5 years. This is another reason why we recommend en bloc PD as superior to partial duodenectomy because partial resection is difficult to obtain extensive lymph node dissection.

Furthermore, based on the KM curve analysis, OS was significantly better in patients with well- or moderately differentiated tumor compared with patients with poorly differentiated tumor. This is probably because of the fact that the histologic type of tumor may affect the lymph node metastasis and prognosis in patients with LARCC as reported by a retrospective study conducted by Saiura et al. [15]. The rate of lymph node metastasis was significantly higher in well-differentiated adenocarcinoma than mucinous or poorly differentiated adenocarcinoma in LARCC (*P* = 0.015) [15]. In our study, only 1 patient (7%) with moderately differentiated adenocarcinoma had node-positive status, whereas all the patients with poorly differentiation adenocarcinoma had node-positive status, and the 3-year OS rates of the two groups (well- or moderately differentiated tumor vs. poorly differentiation adenocarcinoma) were 82% versus 0%.

Molecular markers such as K-Ras, N-Ras, B-Raf, HER2, and MSI play a significant role in the disease prognosis in CRC, and hence, analysis of these biomarkers helps in facilitating proper treatment to the needy patients [30]. In our study, all the patients in MSI-H status survived for > 3 years, while 3-year OS of patients in MSS status was only 35%. OS did not differ significantly between K-Ras mutant and wild-type, BRAF V600E mutant and wild-type tumors nor Her-2. Hence, only MSI was the significant prognostic factor affecting survival.

In FOxTROT trial, preoperative chemotherapy has resulted in significant downstaging of tumor in patients with locally advanced colon cancer compared to postoperative chemotherapy (*P* = 0.04) [31]. Another retrospective study by Arredondo et al. has also shown tumor downstaging (62.5%), R0 resection (100%), and a promising prognosis (median OS of 31 months) in locally advanced colon cancer patients treated with preoperative chemotherapy [32]. Similarly, in our study, the 3-year OS rates was greater in the preoperative and postoperative chemotherapy group (100% and 77.8%) compared with no perioperative chemotherapy group (25.0%). However, these finding need to be confirmed by considering studies with large sample size.

There are several limitations associated with the present study. First, the number of LARCC patients with invasion of duodenum and/or pancreas is low, and hence, the number of participants is small in this study. Large-scale studies may produce more reliable results, nevertheless, our series is one of if not the largest series reported to date. Second, the chemotherapy regimens varied among patients. In the era of advanced chemotherapy, administering the same regimen for a long-term study seems formidable. However, the present study also has several strengths such as this study gathered the largest number of patients, and all the clinical information and follow-up were proved to be accurate. Moreover, histologic-genetic examination was performed in detail, and we were able to build a bridge between the preoperative clinical evidence with histologic-genetic findings and prognosis.

## Conclusions

En bloc RHCPD may result in long-term survival in patients having LARCC with infiltrated adjacent organs. This aggressive approach may help improve the prognosis, particularly in patients with node-negative status. This study also evaluated the prognostic factors for OS and the role of mutational status of several genes on disease prognosis. Long-term and larger-scale studies may produce more reliable results.

## Supplementary Information

**Additional file 1: Table 1.** Clinical characteristics and treatments of the study patients with LARCC. **Table 2.** Pathologic findings and genetic testing in the study patients.

## Data Availability

All data generated or analysed during this study are available from the corresponding author on reasonable request.

## Abbreviations

AJCC: American Joint Committee of Cancer
CI: Confidence interval
CRC: Colorectal cancer
CEA: Carcinoembryonic antigen
CA19-9: Cancer antigen 19-9
CT: Computed tomography
DFS: Disease-free survival
DGE: Delayed gastric emptying
HR: Hazard ratio
LARCC: Locally advanced right-sided colon cancer
MSI: Microsatellite instability
NGS: Next-generation sequencing
OS: Overall survival
PF: Pancreatic fistula
RHCPD: Right hemicolectomy with pancreatoduodenectomy
RC: Right colectomy
SMV: Superior mesenteric vein
