# Supplementary material for: En bloc right hemicolectomy with pancreatoduodenectomy for right-sided colon cancer invading duodenum
Source: BMC Surg. 2021 Jun 29;21:302. doi: 10.1186/s12893-021-01286-0 (PMC8243482; doi:10.1186/s12893-021-01286-0)
Supplement: Supplementary file 1 — Additional file 1: Table 1. Clinical characteristics and treatments of the study patients with LARCC. Table 2. Pathologic findings and genetic testing in the study patients. [file 12893_2021_1286_MOESM1_ESM.docx]

**TABLES**

**Table 1. Clinical characteristics and treatments of the study patients with LARCC**

| **Case** | **Preoperative CEA (ng/mL)** | **Preoperative CA19-9**  **(IU/mL)** | **Comorbidity** | **Operative Time (Minutes)** | **OBL**  **(mL)** | **Blood Transfusion (IU)** |
| --- | --- | --- | --- | --- | --- | --- |
| 1 | 5.0 | 86.4 | A | 410 | 400 | 4 |
| 2 | 7.6 | 228.4 | - | 325 | 300 | - |
| 3 | 14.5 | 2.7 | A | 315 | 500 | 4 |
| 4 | 6.2 | 28.5 | A | 345 | 300 | 2 |
| 5 | 4.3 | 118.5 | A | 390 | 400 | 6 |
| 6 | 3.9 | 192.2 | A | 360 | 200 | - |
| 7 | 0.9 | 12.1 | H | 390 | 100 | - |
| 8 | 6.0 | 3.3 | HT | 300 | 400 | - |
| 9 | 2.1 | 10.7 | A | 365 | 300 | 4 |
| 10 | 1.5 | 11.0 | A | 290 | 200 | 4 |
| 11 | 16.6 | 5.4 | A | 284 | 100 | - |
| 12 | 1.6 | 0.6 | - | 287 | 100 | - |
| 13 | 6.0 | 59.7 | - | 321 | 200 | - |
| 14 | 4.9 | 11.5 | A | 330 | 600 | 6 |
| 15 | 2.4 | 12.6 | - | 222 | 100 | - |
| 16 | 2.2 | 15.8 | A | 330 | 200 | 4 |
| 17 | 2.2 | 18.4 | A | 261 | 200 | 4 |
| 18 | 18.5 | 0.5 | A | 315 | 300 | 6 |
| 19 | 1.8 | 2.7 | A,HT | 240 | 200 | 4 |

A, Aanemia; H, hepatitis; HT, hypertension;

**Table 2. Pathologic findings and genetic testing in the study patients**

| **Patients** |  | **Pathologic Findings** | | | | | **Genetic Testing** | | | | |
| --- | --- | --- | --- | --- | --- | --- | --- | --- | --- | --- | --- |
|  | **Tumor differentiation** | | **Adjacent Organ invading** | **T** | **N** | **M** | **KRAS** | **NRAS** | **BRAF** | **Her-2** | **MSI** |
| 1 | Wel | | Du+Pa | 4b | 0 | 0 | Mutant | W | W | W | MSS |
| 2 | Mod | | Du+Pa | 4b | 0 | 0 | Mutant | W | W | W | MSI-H |
| 3 | Mod | | Du+L | 4b | 0 | 0 | W | W | W | W | MSI-H |
| 4 | P | | Du | 4b | 2b | 0 | Mutant | W | W | W | MSS |
| 5 | P | | Du | 4b | 2a | 0 | Mutant | W | W | W | MSS |
| 6 | P | | Du | 4b | 1b | 0 | W | W | W | W | MSS |
| 7 | Mod | | Du+L | 4b | 0 | 0 | Mutant | W | W | W | MSI-H |
| 8 | Mod | | Du+Pa | 4b | 0 | 0 | Mutant | W | W | Mutant | MSI-H |
| 9 | Mod | | Du+Pa | 4b | 1b | 0 | W | W | W | Mutant | MSS |
| 10 | Mod | | Du | 4b | 0 | 0 | Mutant | W | W | W | MSI-H |
| 11 | Mod | | Du | 4b | 0 | 0 | W | W | W | W | MSS |
| 12 | Mod | | Du+Pa | 4b | 0 | 0 | W | W | W | W | MSI-H |
| 13 | Mod | | Du | 4b | 0 | 0 | Mutant | W | W | W | MSS |
| 14 | Mod | | Du | 4b | 0 | 0 | W | W | Mutant | W | MSI-H |
| 15 | Wel | | Du | 4b | 0 | 0 | Mutant | W | W | W | MSS |
| 16 | Mod | | Du | 4b | 0 | 0 | W | W | W | W | MSS |
| 17 | P | | Du | 4b | 2b | 0 | Mutant | W | W | W | MSS |
| 18 | Mod | | Du | 4b | 0 | 0 | W | W | W | W | MSI-H |
| 19 | Wel | | Du | 4b | 0 | 0 | Mutant | W | W | W | MSS |

MSS, microsatellite stable; MSI-H, microsatellite instability-high; Mod, moderately differentiated; P, poorly differentiated; wel, well-differentiated; W, wild-type
